# The sexual behavior of Tunisian adults during Ramadan: an opinion survey

**DOI:** 10.1192/j.eurpsy.2025.2313

**Published:** 2025-08-26

**Authors:** H. Ktari, B. Amamou, K. Abdessatar, H. Mami, M. Oumaya, R. Bouzid

**Affiliations:** 1 Psychiatry, Fattouma Bourguiba Hospital, Monastir; 2 Psychiatry, Taher Maamouri Hospital, Nabeul, Tunisia

## Abstract

**Introduction:**

The month of Ramadan, a sacred period in the Islamic calendar, is a time of fasting, prayer, and reflection for millions of Muslims worldwide. While the effects of fasting on physical and mental health have been extensively studied, there has been little focus on its specific impact on sexual life, particularly in the Tunisian context.

**Objectives:**

The aim of our research was to study the opinions of Tunisians regarding their sexual behavior during the month of Ramadan.

**Methods:**

This was a retrospective, descriptive, and comparative study conducted through an online survey among Tunisian adults. Data collection was carried out via a self-administered online questionnaire during Ramadan 2024, from March 21 to April 4, 2024. Two reminders were sent and the anonymity of the responses was guaranteed.

**Results:**

Our study included 130 Tunisian adults with a mean age of 28.69 years. The majority of our population resided in urban areas (98.5%), 43.8% were single where 29.2% were in a relationship and both genders were equally represented (53.1% female, 46.9% male). The majority of participants identified as heterosexual (89.2%). During Ramadan, 78.5% of participants fasted, and 77.7% believed sexual activity was permissible during this period. Overall, 44.6% of participants felt that fasting had a negative impact on sexual behavior, 30.7% on sexual desire, and 26% on sexual performance. More than 70% reported that fasting influenced their sexual behavior, primarily in a negative way (44.6%). Among those who perceived an impact (n=93), changes were mostly noted in the timing (77.7%) and frequency of sexual activities (75.5%). Furthermore, 72.3% of participants reported that fasting influenced their sexual desire, with 40.9% perceiving this influence positively. Behavioral factors were identified as the most common cause of these changes (52.1%), followed by religious reasons (27.7%) and societal factors (11.7%). Significant gender differences were observed, with women being more affected by religious factors (p=0.02), while men were more influenced by behavioral factors (p=0.03). Women also reported a significantly greater impact on the frequency of sexual activity compared to men (p=0.012) and perceived a more significant negative impact of fasting on sexual performance compared to men (p=0.06).

**Conclusions:**

Our study indicates that Ramadan fasting significantly affects the sexual lives of Tunisians, driven by sociocultural, religious, and physiological factors. This highlights the need for culturally sensitive sexual education and targeted health policies to ensure accessible and inclusive care during Ramadan.

**Disclosure of Interest:**

None Declared

